# Design and Development of the Brain Training System for the Digital “Maintain Your Brain” Dementia Prevention Trial

**DOI:** 10.2196/13135

**Published:** 2019-02-27

**Authors:** Courtney Campbell Walton, Amit Lampit, Christos Boulamatsis, Harry Hallock, Polly Barr, Jeewani Anupama Ginige, Henry Brodaty, Tiffany Chau, Megan Heffernan, Perminder Singh Sachdev, Maria A Fiatarone Singh, Michael Valenzuela

**Affiliations:** 1 School of Psychology University of Queensland Brisbane Australia; 2 Regenerative Neuroscience Group Brain and Mind Centre University of Sydney Sydney Australia; 3 Sydney Medical School University of Sydney Sydney Australia; 4 Academic Unit for Psychiatry of Old Age University of Melbourne Melbourne Australia; 5 Department of Neurology Charité–Universitätsmedizin Berlin Berlin Germany; 6 School of Computing, Engineering and Mathematics Western Sydney University Sydney Australia; 7 Centre for Healthy Brain Ageing School of Psychiatry University of New South Wales Sydney Australia; 8 Dementia Centre for Research Collaboration School of Psychiatry University of New South Wales Sydney Australia; 9 Neuropsychiatric Institute The Prince of Wales Hospital Sydney Australia; 10 Physical Activity, Lifestyle, Ageing and Wellbeing Faculty Research Group Faculty of Health Sciences, Sydney Medical School University of Sydney Sydney Australia; 11 Jean Mayer USDA Human Nutrition Research Center on Aging Tufts University Boston, MA United States; 12 Hebrew Senior Life Boston, MA United States

**Keywords:** computerized cognitive training, dementia, clinical trial design, older adults

## Abstract

**Background:**

Dementia is the leading cause of disability worldwide, and interventions aimed at reducing the prevalence and burden of the disease are urgently needed. Maintain Your Brain (MYB) is a randomized controlled trial of a multimodal digital health intervention targeting modifiable dementia risk factors to combat cognitive decline and potentially prevent dementia. In addition to behavioral modules targeting mood, nutrition, and physical exercise, a new Brain Training System (BTS) will deliver computerized cognitive training (CCT) throughout the trial to provide systematic, challenging, and personally adaptive cognitive activity.

**Objective:**

This paper aimed to describe the design and development of BTS.

**Methods:**

BTS has been designed with a central focus on the end user. Raw training content is provided by our partner NeuroNation and delivered in several innovative ways. A baseline cognitive profile directs selection and sequencing of exercises within and between sessions and is updated during the 10-week 30-session module. Online trainers are available to provide supervision at different levels of engagement, including face-to-face share-screen coaching, a key implementation resource that is triaged by a “red flag” system for automatic tracking of user adherence and engagement, or through user-initiated help requests. Individualized and comparative feedback is provided to aid motivation and, for the first time, establish a social support network for the user based on their real-world circle of friends and family.

**Results:**

The MYB pilot was performed from November 2017 to March 2018. We are currently analyzing data from this pilot trial (n=100), which will make up a separate research paper. The main trial was launched in June 2018. Process and implementation data from the first training module (September to November 2018) are expected to be reported in 2019 and final trial outcomes are anticipated in 2022.

**Conclusions:**

The BTS implemented in MYB is focused on maximizing adherence and engagement with CCT over the short and long term in the setting of a fully digital trial, which, if successful, could be delivered economically at scale.

**Trial Registration:**

Australian New Zealand Clinical Trials Registry ACTRN12618000851268; https://www.anzctr.org.au /Trial/Registration/TrialReview.aspx?id=370631&isReview=true

## Introduction

### Background

Late-life engagement in cognitively stimulating activities is associated with reduced risk for incident dementia [1]. Computerized cognitive training (CCT) is a specific type of structured cognitive activity that aims to enhance and maintain cognitive performance by means of repeated practice on controlled learning events, targeting specific cognitive processes [2,3]. CCT differs from other types of cognitive interventions by focusing on implicit practice rather than explicit teaching of strategies [4] and has several advantages over traditional pencil-and-paper approaches, chiefly, adaptivity, personalization, flexible administration, and engaging game-like environments [2,5]. Systematic reviews and meta-analyses of randomized controlled trials have established the efficacy of CCT for overall and domain-specific performance in healthy older adults [5], mild-to-moderate Parkinson disease [6], mild cognitive impairment [7], and major depressive disorder [8] in contrast to a lack of efficacy in people with established dementia [7].

There are no established standards for planning and delivering CCT [9]. Design factors such as the content of the training program, training schedules, delivery methods, and combinations with other interventions (eg, physical exercise) vary substantially within and across studies. Literature in this regard suggests that several key design factors may be important for treatment outcomes and fidelity and are briefly reviewed in this paper.

### Targeted Domains

Cognitive effects of CCT tend to relate to the domains trained by the specific program [10,11]: Improvements in untrained tasks (mainly neuropsychological outcome measures) are more likely if the CCT program provided exercises in the same or related (proximal) cognitive domains. Thus, single-domain CCT programs such as those that train only working memory are less likely to lead to meaningful effects beyond the trained domain [5,12]. Since clinical endpoints in older adults comprise global cognitive outcomes, CCT programs typically include a variety of tasks targeting multiple cognitive domains, but the exact composition of domains within programs varies from one program to another [7].

### Training Content

A key design consideration in multidomain CCT is the specific selection of targeted domains and related exercises. There are four major approaches to content design. The first and most common is a fixed schedule, whereby all participants receive the same content across all sessions [13,14]. This design is easy to replicate, but ignores individual differences and therefore may over- or undertarget cognitive strengths and weaknesses at the individual level. Second, the approach taken by most commercial CCT providers as well as in the Neuropsychological Educational Approach to Cognitive Remediation [15] is to allow participants to choose the exercises for each session. This approach may improve subjective outcomes and attitudes toward the program [16], but may limit overall improvement, as users tend to spend more training time on exercises they enjoy and perceive as strengths. Third, some studies use baseline cognitive profiles to guide individual training plans, so that areas of deficit will receive more training time [17]. This approach better addresses individual differences, but ignores domain-specific adaptation, which is the variability of training time required to induce change in the underlying ability [14]. Finally, the most custom-tailored option is to adapt content by setting an initial training plan founded on baseline performance and then changing the composition of exercises at set time points in response to within-training task performance [18].

### Adaptivity

A specific advantage of CCT is the ability to adjust task difficulty and content to individual abilities and progress. Adapting training difficulty is assumed to increase engagement and build skills over time, and adaptive designs tend to be more efficacious than nonadaptive training [18,19,20]. Typical difficulty (“level”) vectors include presentation length, response speed thresholds, number of stimuli, or problem complexity. One particularly useful adaptivity method is the “staircase” algorithm, whereby training difficulty is adjusted during a block, and the level will change after a certain number of consecutive correct or incorrect responses. Some programs change difficulty only between blocks, whereas others implement this within an exercise session.

### Feedback

Feedback is crucial for any learning process and can be an important component that motivates people to engage with the CCT program over time, but its application to CCT is complex and a surprisingly understudied area of work [21]. Most CCT programs will provide feedback for each response within a block (right or wrong), which often assists individuals to develop skills in the specific task. Feedback after blocks may include a temporal (ie, reference to the past performance) or social comparison (ie, reference to others). Other common elements are cumulative scores (eg, “medals” or “brain points”) that convey a sense of progress and may support long-term adherence. Results from previous studies that tried to identify the most effective feedback mechanism report inconclusive results. For example, Burgers and colleagues [22] found that positive feedback was associated with greater motivation to train on the same task in the future, whereas negative feedback increased motivation to train immediately after feedback was given, arguably in order to compensate for performance in the previous attempt, while social comparison decreased motivation overall. Conversely, Katz and colleagues [21] examined the effects of gaming elements such as real-time scoring and scaffolding in children and found that these were distracting and did not lead to better performance compared to neutral training. However, the generalizability of such results to longer-lasting CCT programs in older adults is unknown.

### Delivery Context, Support, and Settings

One of the major advantages of CCT over other cognition-oriented approaches may be the potential to deliver the intervention online inexpensively and at scale. This opportunity, however, has not yet shown sufficient efficacy in the literature. Large trials of home-based CCT reported substantial attrition [23] and frustration [24] as well as low compliance with the training program compared with laboratory-based, supervised training [25]. Furthermore, a comprehensive meta-analysis by Lampit and colleagues [5] found a statistically significant difference between training effects of home-based CCT compared to supervised settings, with the latter estimated to be about three times larger than the former. Novel uses of technology to assist the effective delivery of CT are required; see the paper by Ge et al [26] for a systematic review of this topic.

It has also been proposed that the repetitive nature of the training exercises, which often resemble cognitive tests, limits the potential for engagement and motivation of participants [27]. Gamification of CCT exercises has been proposed as a potential method of maximizing participants’ interest; however, this has not been extensively studied [28]. In the broader literature, it is known that individuals respond best and engage with learning and training when they are intrinsically motivated to do so [29]. Home-based training may be less sensitive to personal differences and thus unable to provide specific motivational cues. Supervision may therefore be important for maintaining adherence by adding a human element to training, motivational support to complete difficult challenges, and problem solving for information technology issues. However, supervision in the present scenario is labor intensive and not scalable to a public treatment at large.

Another potentially crucial aspect of CCT delivery that has not been explored systematically is the consideration of within-session sequencing of different CCT exercises. As described above, it is likely that motivation and engagement are fundamentally linked to an individuals’ desire to engage with training and thus maximize the potential for cognitive improvements [30,31]. Exploring novel methods of maximizing individuals’ ability to engage with exercises that target the most difficult exercises (ie, their weaknesses) is therefore important. To our knowledge, no study has assessed this fundamental design element.

Finally, one of the most important factors in long-term engagement with *any* behavioral intervention is building a community of practice [32], a concept co-opted from organizational theory [33,34]. Joint enterprise (improvement of brain health), mutual engagement (training attendance and adherence), and shared repertoire (learning and mastering the software and exercises) are the core self-sustaining features of a communal practice [35]. These factors are easier to address in center-based CCT, since trainees tend to meet with other trainees in the laboratory or facility, cross-validate each other’s reason for being there, and engage with and receive instructions from trainers or research staff. In contrast, with home-based CCT, individuals have a high risk of feeling isolated, lacking support, or not understanding the relevance of the activity to the “real world.” This is yet another potential reason for the high rate of attrition and low treatment fidelity reported in many home-based CCT studies.

### The “Maintain Your Brain” Digital Health Trial

In the following section, we will outline how these design considerations have been addressed in the digital Brain Training System (BTS), which is one of four intervention “modules” within the Maintain Your Brain (MYB) trial (trial registration: Australian New Zealand Clinical Trials Registry, ACTRN12618000851268) [36]. MYB is the largest online cognitive decline-prevention trial to date and has recruited 6200 Australians aged 55-77 years with multiple dementia risk factors but no dementia diagnosis. Participants were recruited from the Sax Institute’s 45 and Up Study [37]. Up to four preventative lifestyle-based modules can be administered depending on the person’s individual risk factor profile: the BTS module for those with an inactive cognitive history or current lifestyle, a physical exercise module for participants who are physically inactive or have chronic diseases/risk factors for dementia known to benefit from exercise (eg, diabetes, hypertension, and frailty), a nutrition module for those reporting dietary intake that does not indicate adherence to a Mediterranean-type cuisine or those who have chronic diseases/risk factors for dementia known to benefit from this type of diet (eg, obesity, cardiovascular disease, and excess alcohol consumption), and a stress- and depression-management module for those with chronic stress or current anxiety/depression-based symptoms; see Heffernan et al [36] for more details on the criteria. Each module is administered sequentially as a 10-week high-intensity block (ie, the maximal 4-module intervention lasts 4 × 10 weeks, although there may be short breaks in between modules), transitioning to monthly booster sessions for the remainder of the 3-year follow-up. Participants allocated to the control group will complete basic tasks such as video quizzes on the MYB platform, instead of completing CCT.

Readers are directed to the trial protocol [36] for further details on all outcomes of the trial. Briefly, the primary outcome will be the change in cognition from baseline to 3 years, as assessed by the MYB cognitive test battery. A number of secondary outcomes will also be assessed to determine the real-world relevance of any improvements in cognitive testing. The following factors are most important to the above-described cognitive module: differences in the occurrence of incident dementia and changes to assess dementia risk [36].

### Aims

Our primary focus is to describe how the novel BTS aims to maximize CCT efficacy in the context of a large-scale, population-based, publicly funded trial with necessary resource restrictions. Several innovations are introduced in this paper, including functionalities to enable online supervision and promotion of a social community as well as our novel “sandwich” algorithm that allows for the principled selection and scheduling of CCT exercises both within and between sessions. This information will be critical to informed interpretation of MYB findings, when available, and may prove useful to researchers conducting similar interventions in the future.

## Methods

### Overview of the Brain Training System Architecture

**Figure 1:**
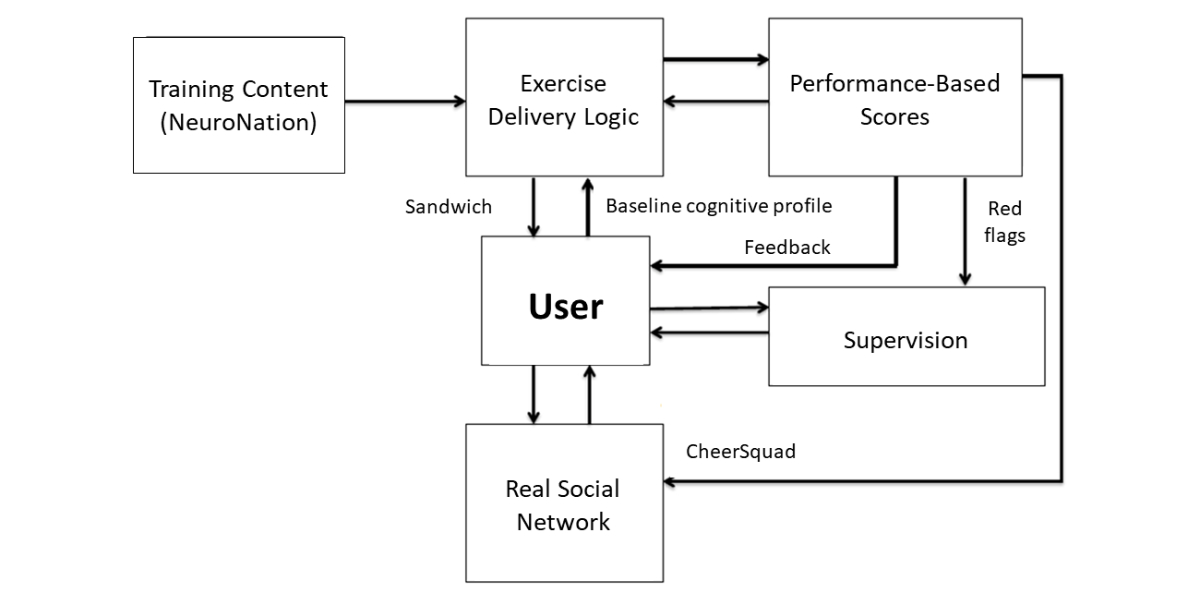
Functional architecture of the Brain Training System (BTS) where the participant is the central focus of activity. For any given session, BTS chooses the exercise based on the "sandwich" algorithm that responds to baseline cognitive profile and ongoing training performance and determines whether a particular exercise is delivered at the beginning, middle, or end of a session. Exercises were provided by our industry partner, NeuroNation. Performance-based scores are used to graphically feedback results to users, update their cognitive profile, and alert online trainers of users who most need support. Finally, real-world support is sought from the user’s network of family or friends in order enhance training adherence, motivation, and experience.

The implementation of CCT involves much more than simply providing a set or sequence of disembodied cognitive exercises. The process is illustrated in Figure 1, where the user is at the center of our system architecture. Exercises have been provided by our collaborating partner, NeuroNation (Berlin, Germany), as a set of 34 stand-alone exercises with their own internal logic and tunable parameters. Sequencing and streaming of these exercises in a user-customized way is a challenge because it requires filling out a matrix of 30 (sessions) × 17 (exercises/session) = 510 exercise slots.

The BTS introduces a novel approach to CCT exercise delivery using logic built around the participant’s initial cognitive profile, which is then updated during the evolution of their training process (detailed further below in terms of the “sandwich”). This logic therefore governs which exercises appear within a given session as well as the order of appearance in that session.

Next, a scoring system based on within-exercise performance was developed, which allows for comparable scores across exercises that are summated at the cognitive domain level. These scores were used for three main purposes: (1) to interact with the exercise logic algorithm to update the user’s cognitive profile during the training process, (2) to provide performance feedback visually to the user, and (3) to automatically identify user engagement, compliance, or adherence issues for supervisory redress using the “red flag” system.

As mentioned above, the quality of supervision is a major determinant of the efficacy of CCT. Because MYB intended to recruit several thousand participants, individual one-on-one supervision, even online, was not feasible or desirable due to the intent of scalability. Our approach was therefore to triage our supervisor’s interaction with users based on need, quantified by aggregation of red flags or user tickets. Supervision involved a combination of online chat messaging, emails, telephone calls, or Skype communication including screen sharing, if required, to provide live feedback on exercise engagement.

Finally, the fifth innovation in BTS was to enmesh the user’s training experience with their real-world social network. This was accomplished with the Training with Friends functionality described below.

### Information Technology for the Brain Training System

The BTS depicted in Figure 1 is not a standalone system, but paired with the main MYB digital system that manages participant data and delivery of online modules. This loosely coupled architecture between MYB and BTS lends itself to ensuring seamless handover of information and that the load of one system does not adversely affect the other. To assist in scalability, BTS has been deployed with a horizontal scaling strategy, should the load on the system become too large for one server.

The PHP framework Laravel [38] was employed to implement BTS and utilizes the model–view–controller [39] architectural pattern. RESTful application programming interface Web Services [40] have been constructed to allow for third-party systems to interact with BTS. Content is consumed and structured such that logic can be applied to drive the participant’s journey and exercise assignment through the system. MariaDB is utilized as the database for all data storage and retrieval.

A participant accessing BTS via MYB invokes a URL redirection (passing through obfuscated and encrypted identifiers) and is prompted to continue with their next available exercise. All exercises are delivered as Shockwave Flash Movie (.swf) files, and the system has embedded these exercises within the same web user interface to provide a seamless user experience. At the completion of each exercise, results are stored within BTS, and these data update the evolving cognitive profile of the participant. In addition, results are sent to the main MYB system via a RESTful application programming interface Web Service, so that the main MYB system aggregates all data across all modules. The BTS then assesses the session state and manages the next exercise or prompts the user that there are no further exercises to complete for that session.

### Cognitive Domains and Content Delivery by the “Sandwich”

Measurement of baseline cognitive abilities and subsequent within-training improvement, along with classification of CCT exercises, correspond to seven cognitive domains: “Verbal Executive,” “Verbal Memory,” “Visual Executive,” “Visual Memory,” “Visual Attention,” “Speed,” and “Working Memory.” A table of the MYB online cognitive tests and BTS exercises with corresponding cognitive domains is presented in Table 1.

Cognitive domains are used to build a cognitive profile, linking cognitive testing with CCT exercises. Classification was determined by consensus across the clinical authorship team. Note that many exercises share some cognitive elements, and classification was therefore based on the predominant and unique cognitive skills required for a given exercise. All exercises were provided by our collaborating commercial partner, NeuroNation. Cognitive testing was accomplished using a combination of an in-house developed LOGOS test and specific subtests from Cambridge Brain Sciences and CogState. CCT exercises are ideally completed across three sessions per week, translating to a module of 30 training sessions over 10 weeks. Each session lasts approximately 45 minutes and comprises 17 exercises. If participants miss a session, the allocated session remains available with no new sessions triggered (ie, sessions cannot be skipped) on the MYB platform until completed or the end of the 10-week intervention module (whichever is sooner). During postmodule follow-up that will last up to 3 years, booster exercises will be offered once a month.

**Figure 2:**
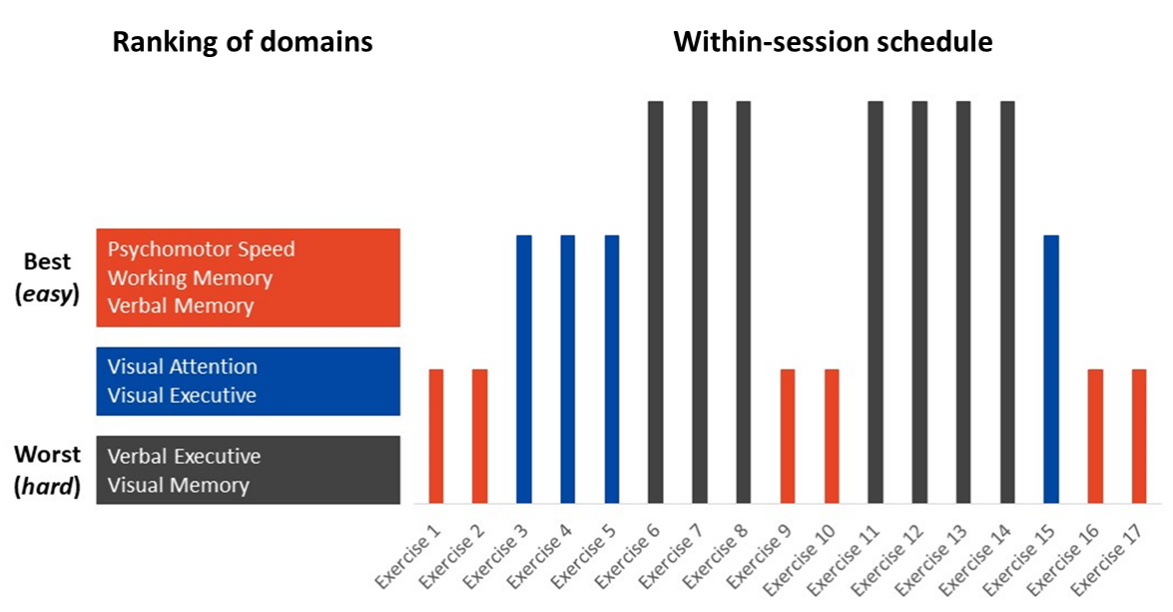
Example of how an individual session of training is formalized using the “sandwich” algorithm. Red represents cognitive domains of strength, grey represents cognitive domains of weakness, and blue represents cognitive domains at the mid-level performance for the user. Cognitive training exercises that correspond to these domains will then be presented one by one. The first two exercises will be in a domain of strength (ie, easier to complete), the next three in the middle domain, the following three in an area of weakness (difficult to complete), and so on (ie, each session, an individual completes six easy, four medium, and seven hard exercises). Thus, the beginning, middle, and end of each session will feature the "easiest" exercises for that individual.

The delivery of these exercises within BTS has been designed to maximize benefits to participants. The focus of this content delivery is the “sandwich” that is based on a novel insight around exercises anticipated to be “hard,” “medium,” or “easy” by participants. The first step in designing the sandwich (Figure 2) is to create a cognitive profile of the participant at baseline. Eight CCTs make up the cognitive profile (Table 1). This includes the MYB Cognitive Battery plus LOGOS (an in-house designed verbal memory measure that is completed over the phone via automated voice recognition). These eight tests have been selected to correspond to the seven cognitive domains described in Table 1. After completion of this battery, an estimate of the participant’s strengths and weaknesses is possible. Standardized z-scores are created by comparing the individual’s performance on each test to normative values collected during our pilot trial. Following this, cognitive domains can be ranked in order of strengths (highest comparative z-score) to weaknesses (lowest comparative z-score). Once this cognitive profile is created, the corresponding CCT exercises can be classified as “easy,” “medium,” or “hard” based on the domains they load upon (Table 1). For example, a CCT exercise tapping into a domain that is an individual’s strength would be considered easy.

With this information, the initial sandwich is created and used for sessions 1-12. Each session will contain six easy, four medium, and seven hard exercises Subsequent sandwiches used in the sessions 13-18, 19-24, and 25-30 will adapt to reflect the participant’s performance on the CCT exercises from the previous session range. Therefore, the cognitive profile originally based on baseline MYB Cognitive Battery will reflect performance on CCT exercises in comparison to the other participants in the trial. Subsequently, the cognitive profile will be adjusted based on the degree of improvement (or lack of) on specific exercises aggregated at the domain level. This therefore allows for adaptivity of content based on an individual’s trajectories and responses to training. The sandwich will be refreshed at weeks 12, 18, and 24.

The training sessions consist of specially selected and integrated exercises from NeuroNation, a German brain-training software company. Each of the 34 training exercises were specifically chosen by the multidisciplinary MYB team to correspond to one of the seven cognitive domains of interest (Table 1). Although each of these exercises have been categorized as targeting a specific cognitive domain, as with most cognitive training exercises, they are inherently multidomain and may also target other domains to a degree. For example, regardless of the classification, many of the memory exercises may also train aspects of speed and attention. Although the domains chosen for training and exercise sequencing within a given session are responsive to the user’s current cognitive profile, where multiple exercises that meet these specifications are available, the choice is pseudorandom.

**Table 1:** Cognitive domains and corresponding training exercises and tests.

Cognitive domain	Cognitive training exercise	Assessment of domain
Verbal Memory	Memory interrupted, Memo pair, Verbal learning	LOGOS
Visual Attention	Eagle eye, Clockwise, Memobox, Quick count, Quick switch	Cogstate - Identification
Visual Memory	Path finder, Path finder reverse, Restorer, Focus master, Polaroid picture, Symbolism, Turnabout, Reflector	Cogstate - One card learning test and Cambridge Brain Sciences - Paired associates
Verbal Executive	Word craft, Scrambled words, Domino word, Password	Cambridge Brain Sciences - Grammatical reasoning
Visual Executive	Plastic puzzle, Solitaria, Escalator, Color craze, Rotator, Form fusion, Missing link	Cambridge Brain Sciences - Spatial search
Working Memory	Parita speed, Form fever, Mixed memories	Cogstate - One-back test
Processing Speed	Split second, Flash glance, Form fever speed, Turning tables, Alphabet soup	Cogstate - Detection

### User Performance

User performance is critical for several reasons. First, these data are used to construct a feedback graph for users, which illustrates how they are performing relative to other users. Although there is no consistent empirical evidence on how to best provide feedback in CCT studies, our team’s clinical experience delivering CCT interventions across a number of populations suggests that some trainees are motivated to reach a minimum level or “standard,” while others are motivated to be the best. We have thus implemented a dynamic bar graph that visualizes how the participant is tracking in terms of performance on the seven cognitive domains, with a target zone representing the top performing 25%. It was our estimation that this graph will motivate those aiming to be at the top without demotivating those who are performing at lower levels. Second, participant performance data are also used to update the user’s cognitive profile and therefore sandwich algorithm. The third function is to automatically monitor compliance, adherence, and treatment fidelity as well as to identify participants who may be struggling to understand or appropriately complete the exercises. This is further described in the section “System-Initiated Flags” below.

### Online Supervision

#### The Brain Training System

As discussed above, home-based CCT can be prone to participant attrition and frustration. In order to ensure participants are able to complete their allocated training with minimal frustration and dropout and to maximize potential benefit of the intervention, BTS was designed such that participants could interact with online trainers. The role of the trainer is to use online messaging, Skype video-conferencing, and phone calls to ensure participants have completed each task correctly and that queries or issues that arise are dealt with. BTS allows three ways in which trainers and trainees can interact.

#### System-Initiated Flags

In order for participants to stay on track and complete their allocated tasks correctly, BTS was designed to automatically create a “flag” if poor performance is detected. Poor performance was defined any of the following scenarios: (1) if the participant provides more incorrect than correct responses in an exercise, (2) if the participant scores zero correct answers in an exercise, and (3) if the participant fails to score above a predefined level or score on that task. These flags are expected to occur more frequently at the outset of training and are sensitive to users who have not understood the basic requirements of the task. In addition, a “decrease in performance” flag was created for a participant who, on two *consecutive* attempts of the same exercise (ie, across sessions), performs ≥10% worse on the second attempt than on the first attempt. This flag is sensitive to participants struggling with increasing difficulty. The aforementioned flags are grouped as “red flags.” A separate group of flags are triggered to advise trainers and participants that adherence is not adequate. This “adherence flag” is created if participants take longer than 90 minutes to complete a training session or are absent for 72 hours between sessions. Adherence flags are visualized in BTS as “orange flags.” Once three orange flags have been created, this system automatically produces an adherence red flag, notifying trainers of a more significant and persistent problem.

When a red flag occurs, it raises a “ticket” in BTS that must be responded to by a trainer. All trainers are alerted via email when a flag (or user-raised ticket, discussed below) is created. A trainer can take ownership of the ticket or assign it to another available trainer. This function allows escalation of tickets to different members and aids in managing rosters with multiple trainers. In addition, any red flag automatically triggers an email to the participants with a link on how to contact a trainer if they need help and tips for avoiding red flags in the future. Automatic emails are capped at 1 every 72 hours to prevent participant overload.

#### User-Initiated Tickets

**Figure 3:**
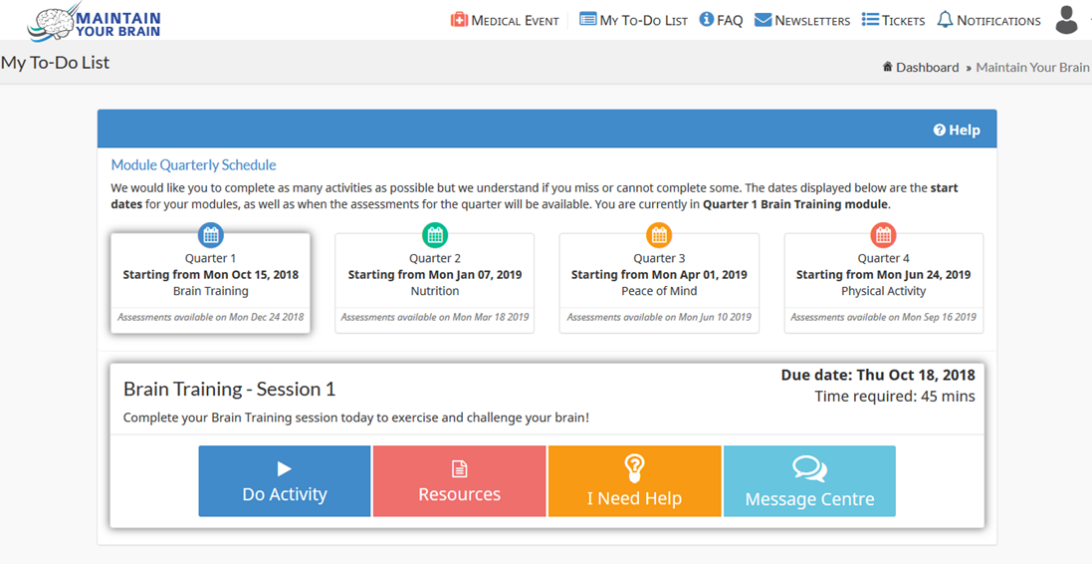
Screenshot of activity list from the participant's view. This centralized user area lists all required activities including outcome tests and training exercises.

**Figure 4:**
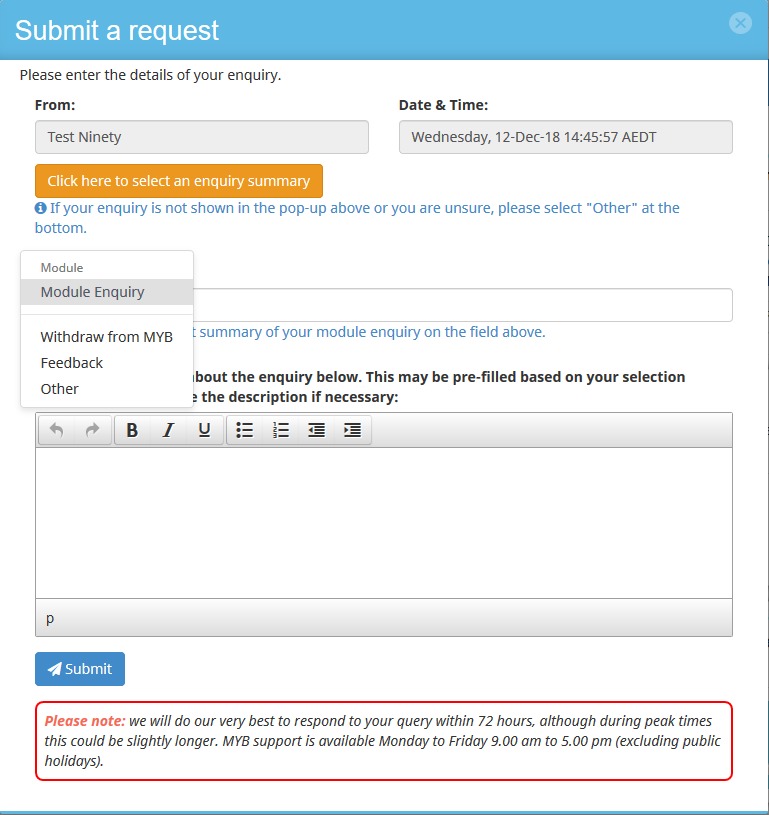
Screenshot of the participant's view for submitting a help request. Participants can directly contact trainers for assistance at any time, by simply selecting "I need help" from their homescreen.

A user can create a ticket themselves by either clicking on the “I need help” button on their home screen or on the “message centre” button (Figures 3 and 4). This provides participants with a list of common issues or the participants can type in their own details. This form of message sends an email to all trainers, so that they can log in and assign the ticket. The assigned trainer will subsequently receive email notifications of any new related messages. The message center is open to all trainers for viewing general issues.

#### Online Trainers

**Figure 5:**
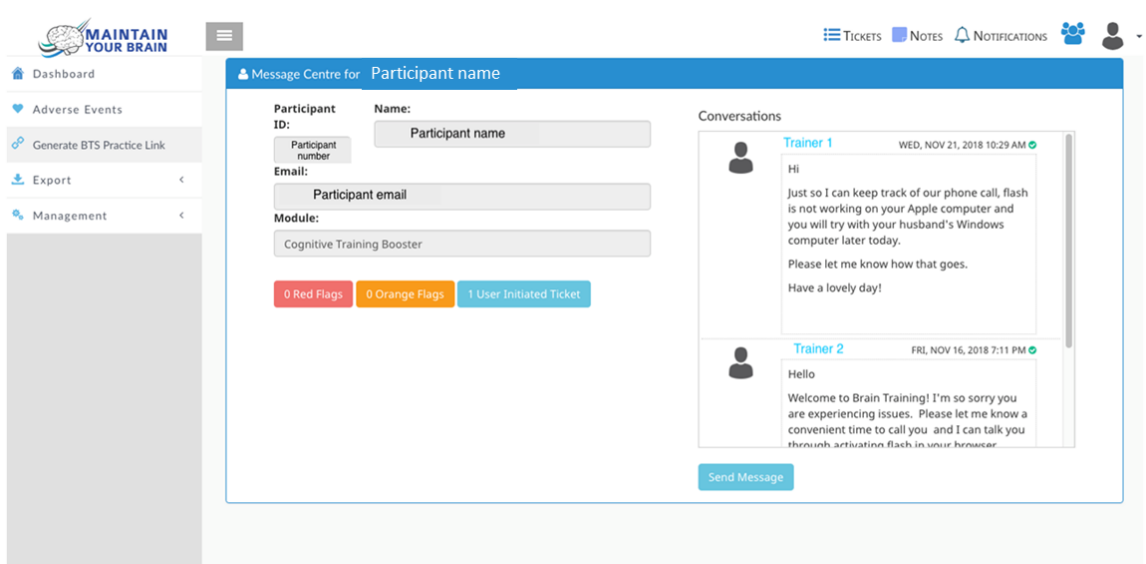
Screenshot of the message center from the trainer's view. Trainers receive messages from participants and can respond using a simple chat format as seen on the right or organize to communicate via email, Skype, or telephone.

Online trainers contact participants (either via the message center, Skype, or phone call) to resolve issues that are raised through the ticketing system. Trainers can drill down at the participant’s specific performance history that triggered a flag and use their discretion to the level of help that the participant may require (eg, a message reminding them of instructions or a phone call to resolve a technical issue). A trainer can respond to each individual ticket (if the issue is task specific), a set of tickets via the message center (if the issue seems to be more general), or an individual help request (Figure 5).

In terms of trainer work flow, user-raised tickets are generally dealt with first. The second priority is participants with the most accumulated red flags. This may result in a phone call/Skype (eg, if a participant is failing to make any responses or any correct responses, it appears as a technical error or a lack of understanding) or a message (eg, if it is for one particular task and written instructions are deemed useful).

Trainers are allocated shifts on a roster with approximate 9 am to 5 pm coverage, 5 days a week. If a trainer is unable to resolve a ticket during his/her shift, he/she can pass on the ticket to the next scheduled trainer. This will give the new assigned trainer a notification when he/she logs into the system (as well as an email). Trainers can allocate a ticket to someone else to deal with (ie, the trainer, manager, or someone in a different area of the specialist MYB team, such as an information technology specialist).

Standard trainer strategies when working with participants includes coaching techniques such as encouraging phrases at the beginning and end of the interaction and dealing with the issue in the middle of the interaction. Trainers are able to generate practice links for participants in order to guide them through the exercise during the phone/Skype call. During our pilot trial, the most common issues have been participants not using the correct internet browser or software, not understanding of the task instructions, or not able to locate the instructional video, all of which were easily resolved by the trainers.

### Real-World Community of Support

A key aim of CCT is to maximize engagement and motivation levels. Given that evidence suggests direct reimbursement for effort is less likely to boost engagement than intrinsic motivation [41], we developed a functionality whereby participants’ family or friends can provide positive reinforcement and encouragement to increase a participants’ sense of achievement, pride, and desire to continue. When participants start the 10-week module, they have the opportunity to list up to five friends/family members to create their “CheerSquad.” These individuals are first emailed an invitation to take up their supporting role and then sent automatic emails when the participant reaches a training milestone. On these occasions, the friends/family members are asked to directly contact the participant and provide personal encouragement and support. Thus, through BTS, participants are designed to receive regular positive feedback from those they feel connected to in order to help maximize motivation and long-term program engagement.

### Open-Access Research Platform

The BTS was created and developed specifically for the MYB trial based on public funding from the National Health and Medical Research Council of Australia. Our intent is therefore to make it as freely available as possible to the international research community. To facilitate this, BTS will be accessible on a cost-recovery basis to verified researchers who have public funding and where the research project does not have a commercial interest, funding, or purpose. Commercial enterprises or commercially funded research projects can also apply for access to the BTS on a non-exclusive fee-for-license basis. Note that users will need to come to their own arrangement with NeuroNation to utilize the company’s CCT content for their particular research project; alternatively, they may substitute the BTS with alternate CCT content using their own information technology expertise and resources.

## Results

The MYB pilot was performed from November 2017 to March 2018. We are currently analyzing data from this pilot trial (n=100), which will make up a separate research paper. The main trial was launched in June 2018. Process and implementation data from the first training module (September to November 2018) are expected to be reported in 2019 and final trial outcomes are anticipated in 2022.

## Discussion

Development of the BTS module and wider MYB platform has been a complex process. It has been designed from the ground up by a multidisciplinary team of a system architects, platform design specialists, software engineers, and contract developers in collaboration with cognitive training and clinical researchers as well as third-party partners to meet the specific needs of MYB. The process of developing the design scope, technical specification, and final structure of BTS took about 1 year and delivery, debugging, and pilot testing the system took another year, including several iterations. The key challenges thus far included delivery of BTS within the budgetary constraints of this publicly funded research; algorithmically formalizing optimal processes for effective trainer-participant interactions, performance tracking, and content delivery; complexities associated with seamless integration with external information technology systems as well as the wider MYB platform; the variability of end user-computing environments including operating systems and internet browsers; use of FLASH video–based content that is increasingly unsupported by modern browsers; development of new CCT exercise content to target verbal memory; and the design, validation, and implementation of a novel automated test of verbal memory. The primary limitation of the platform design is that mapping of cognitive tests and exercises is based on consensus estimates of the most relevant cognitive domain and cannot account for the inherent multidomain nature of such tasks. Additionally, this platform introduces a number of novel factors (the sandwich, online trainers, feedback, and social support) that will not be independently assessed for efficacy.

In this paper, we have presented the design of a scalable system for delivery of CCT based on the best evidence to date. BTS is a key intervention module within the MYB trial that is anticipated to be the largest digital health intervention for cognitive decline and dementia prevention so far [36]. To the best of our knowledge, our CCT technology is unique and promises to increase our understanding of how to implement and facilitate effective training for older adults at home. This is a crucial unmet need, and we hope it will contribute to a reduction in the occurrence of dementia and cognitive decline in the community.

## Abbreviations

CCT: computerized cognitive training
MYB: Maintain Your Brain
BTS: Brain Training System
